# In Response

**DOI:** 10.4269/ajtmh.14-0479

**Published:** 2014-12-03

**Authors:** Douglas C. Heimburger, Catherine Lem Carothers, Sten H. Vermund

**Affiliations:** Vanderbilt Institute for Global Health, Fogarty International Clinical Research Scholars, Support Center at Vanderbilt University, Nashville, TN. E-mails: douglas.heimburger@vanderbilt.edu, catherine.lem@vanderbilt.edu, and sten.vermund@vanderbilt.edu

Dear Sir:

We appreciate the interest of Drs. Benziger and Gilman in the Fogarty International Clinical Research Scholars and Fellows (FICRS-F) Program.^1^,^2^ We share their appreciation for the value of the program's year-abroad mentored research opportunities for both postdoctoral fellows and doctoral students. We wish to provide further information and to clarify points raised. The editorialists note that there are fewer slots for doctoral students in the Fogarty International Center's (FIC) redesigned program, Global Health Program for Fellows and Scholars (GHPFS), in which leadership and coordination are divided among five consortia of four institutions each.^2^ Indeed, FICRS provided unique opportunities for doctoral students, and these are now limited in GHPFS. Although the FICRS Program's “twinning” of United States doctoral students with low- and middle-income country (LMIC) trainees added value to both trainees' experiences, GHPFS consortia can choose to twin or otherwise link their United States trainees with LMIC counterparts if they wish. We also agree that the pre-deployment Program orientation held on the National Institutes of Health (NIH) campus has been an extremely effective component of the FICRS-F Program. Grant transition precluded orientation in 2012, but a 1-week annual orientation has been held since 2013. Orientation offers networking opportunities with attending mentors and other trainees, exposure to multiple NIH global health programs and scientists, and introductory training in quantitative and qualitative research methods (19 contact hours in the July 2014 orientation). Finally, the editorialists are concerned that opportunities for trainees to return to their international sites and to present their results at international meetings are now lost; in fact, they remain, subject to the discretion and resources of the consortia.

We are engaged in a multi-year project to report on the FICRS-F program's outputs and outcomes.^4^–^7^ We are monitoring FICRS-F alumni publications, grants, career positions, networks, and retention in global health and in research, and their reflections on factors that had pivotal impacts on them. We are comparing early versus late trainees, doctoral versus postdoctoral trainees, United States versus LMIC trainees, and trainees who were formally twinned versus those who were not twinned. To follow alumni, we use social media, e-mail, communications with their mentors, and interactions at professional meetings. Relying on alumni to provide regular updates is unrealistic, and we look to the FIC to ensure that tracking is supported to permit long-term analyses of the unique impacts of FICRS-F and other research training programs.
